# Converting CO_2_ to formic acid by tuning quantum states in metal chalcogenide clusters

**DOI:** 10.1038/s42004-023-00851-3

**Published:** 2023-03-21

**Authors:** Turbasu Sengupta, Shiv N. Khanna

**Affiliations:** Department of Physics, Virginia Commonwealth University, Richmond, VA 23284-2000 USA

**Keywords:** Catalytic mechanisms, Electronic structure, Electronic properties and materials, Reaction mechanisms

## Abstract

The catalytic conversion of CO_2_ into valuable chemicals is an effective strategy for reducing its adverse impact on the environment. In this work, the formation of formic acid via CO_2_ hydrogenation on bare and ligated Ti_6_Se_8_ clusters is investigated with gradient-corrected density functional theory. It is shown that attaching suitable ligands (i.e., PMe_3_, CO) to a metal-chalcogenide cluster transforms it into an effective donor/acceptor enabling it to serve as an efficient catalyst. Furthermore, by controlling the ratio of the attached donor/acceptor ligands, it is possible to predictably alter the barrier heights of the CO_2_ hydrogenation reaction and, thereby, the rate of CO_2_ conversion. Our calculation further reveals that by using this strategy, the barrier heights of CO_2_ hydrogenation can be reduced to ~0.12 eV or possibly even lower, providing unique opportunities to control the reaction rates by using different combinations of donor/acceptor ligands.

## Abstract (web summary)

Finding an efficient catalyst to convert CO_2_ into useful products is a challenging problem. Here, the authors use first-principles calculations to show how the attached donor/acceptor ligands on the Ti_6_Se_8_ cluster can be utilized to design effective catalysts for CO_2_ hydrogenation to formic acid.

## Introduction

The rapid increase of the CO_2_ level in the atmosphere has become a serious concern to mankind in recent times^1,2^. One of the direct solutions to the problem is utilizing porous or mesoporous adsorbents as a medium for CO_2_ capture^3^. However, the high cost associated with storage and transport restricts such procedures from large-scale industrial applications^4^. As an alternative, the conversion of CO_2_ into useful chemicals represents a relatively cost-effective strategy^4–6^. Aside from reducing CO_2_ concentration in the atmosphere, the converted products can also be utilized as a resource for value-added chemicals. Among the assortment of chemicals that CO_2_ can be chemically converted into, formic acid (FA) represents a compelling choice for a multitude of reasons^7–9^. As a chemical, formic acid is extensively used as feedstock material and can also be transformed into value-added products with relative ease. As an energy-dense material, it can also be used as an alternative to fossil fuels, thereby being effective in reducing the carbon footprint. Additionally, having a high volumetric hydrogen density, it also has immense potential as an effective hydrogen storage vector^8^. At room temperature, formic acid is a low-toxic liquid; therefore, the storage and transportation of formic acid are significantly cost-efficient. As a result of these advantages, a significant amount of worldwide CO_2_ conversion is now performed in the form of formic acid, and the net conversion quantity is rising at a rapid pace every year^4,8^.

One of the major challenges of converting CO_2_ to formic acid is the inherent inertness of CO_2_. Being thermodynamically and kinetically stable^10^, CO_2_ hydrogenation without an effective catalyst is a difficult task under normal conditions. Nowadays, a range of homogeneous and heterogeneous catalysts are available for CO_2_ hydrogenation, and the reaction can be carried out thermochemically or electrochemically as required^11–18^. One of the focuses of this current paper is to evaluate the potential of the metal chalcogenide clusters (e.g., Ti_6_Se_8_) toward thermochemical CO_2_ conversion to formic acid. In recent times, ligated metal chalcogenide clusters have gained considerable attention due to their high stability and ease of synthesis in a solvent medium^19–23^. These stable ligated clusters can also be assembled as ionic solids with complementary units like fullerenes. In the past decades, significant numbers of such clusters and assemblies have been experimentally synthesized by Roy, Nuckolls, and coworkers^20–25^. In such solids, the ligand-protected cluster cores usually act as charge donors, whereas the fullerene moieties are charge acceptors, resulting in extended three-dimensional ionic crystals that are similar in structure to CdI_2_ or NaCl. Incidentally, it was also shown^26–33^ that within such solids, the ligated clusters maintain their chemical identity, and the attached ligands control the donor/acceptor characteristics of the clusters, providing a unique opportunity to predictably alter the properties of the whole superstructure altogether.

Historically, ligands have always played a pivotal role in cluster chemistry^34–37^. Apart from protecting the sensitive cluster core or preventing a cluster from coalescence or leaching, they can also be utilized to electronically stabilize a metal cluster by filling up the valence shell to the nearby magic number. In some rare instances, it was observed that ligands could also enhance the reactivity of a cluster via the formation of localized active sites or donor-acceptor pairs^38–41^. In that regard, the effect of ligands on the electronic structure of the metal chalcogenide cluster is observed to be unique. It has been shown^26–33^ that by controlling the number and type of the attached ligands, one can transform a metal chalcogenide cluster into a strong electron donor or an acceptor by shifting the whole electronic spectrum without altering the valence shell configuration. For example, we have shown that strong σ-donor ligands like phosphines induce an upward shift of the electronic spectrum, whereas a shift in the reverse direction is noticed for strong π-acceptor ligands like CO. The overall effect of the ligands in such cases can be conceptualized as a Coulombic well that surrounds the cluster core and thereby influencing the discrete energy levels of the cluster.

In the present paper, we have considered Ti_6_Se_8_ as a model cluster catalyst and investigated its catalytic potential toward formic acid synthesis via CO_2_ hydrogenation. Our calculation of the minimum energy pathway reveals that in comparison to other reported catalysts, the unligated Ti_6_Se_8_ cluster is a really good catalyst for CO_2_ hydrogenation with significantly low barrier heights ranging from ~ 0.3-0.4 eV^42–44^. However, what sets the Ti_6_Se_8_ cluster apart from any conventional catalyst, is not the lower barriers for hydrogenation but rather the dependence of the barrier heights on the attached ligands. Controlling the rate of a chemical reaction has always remained an elusive goal for chemists and materials scientists. However, apart from a few exceptional examples, reports of such achievements are rare. Among the notable examples, controlling reactivity by microwave irradiation^45^ is observed earlier for an electron transfer reaction. Recently, Pan and Liu^46^ have shown an interesting example where control of chemical reactivity is achieved by Fermi-coupled vibrational states. In this work, we show that a similar goal can also be achieved by ligated Ti_6_Se_8_ clusters simply by varying the number and type of the attached ligands. It is observed that attaching π-acceptor ligands, e.g., CO, to the cluster results in an increase of hydrogenation barriers compared to pristine Ti_6_Se_8_, whereas σ-donor ligands like PMe_3_ reduce the barriers for the reaction. Thus, by selectively controlling the ratio of attached acceptor and donor ligands to the cluster, one can increase or decrease the barrier heights for CO_2_ hydrogenation in a stepwise manner. Our calculation further indicates that by attaching only 3 PMe_3_ ligands to the cluster, one of the CO_2_ hydrogenation barriers can be reduced to as low as 0.12 eV. Investigation of the electronic structure of the ligated clusters proves that the alteration of the barrier heights is due to the ligand-induced shift in the electronic levels. The upward shift of the electronic levels by PMe_3_ facilitates the electron transfer as well as energetically destabilizes the cluster-hydrogen bond and assists the release of H atom(s) and, thereby, the reaction. An opposite effect hinders the reactivity when one or more CO ligands are introduced. Thus, we have shown that apart from protecting the cluster core, ligands can also be utilized as a tool for controlling the reactivity of the cluster. In regard to that, our current investigation offers a simplistic, cost-efficient, and easily achievable approach to the problem.

## Results

### Calculation of the reaction pathway of CO_2_ → HCOOH conversion on the Ti_6_Se_8_ cluster

**Fig. 1:**
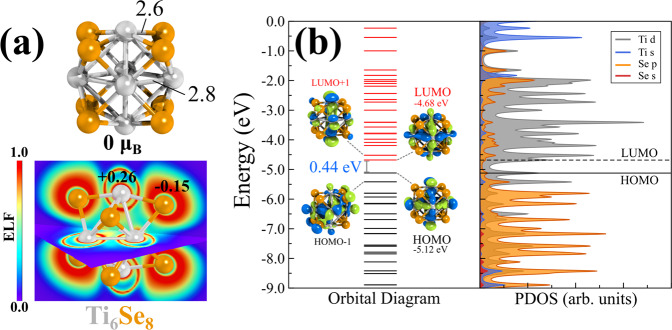
Structure and electronic properties of Ti_6_Se_8_ cluster. **a** Ground state optimized structure of Ti_6_Se_8_ cluster (bond distances are in Å) and the contour plot of the electron localization function (ELF) along with the Hirshfeld charges on the Ti and Se atoms (**b**) the molecular orbital (MO) and the projected density of states (PDOS) diagram of Ti_6_Se_8_ cluster. Isosurfaces of a few important orbitals are also shown.

To investigate the catalytic potential of the metal chalcogenide cluster toward CO_2_ hydrogenation, we have chosen the Ti_6_Se_8_ cluster as our template catalyst. In previous investigations, titanium-doped nanoparticles and surfaces have shown remarkable promises and performances toward CO_2_ reduction reactions^47–50^. Our choice of Ti_6_Se_8_ cluster is influenced by such experimental and theoretical results. It is noteworthy that although the specific Ti_6_Se_8_ cluster reported in this paper is not yet reported experimentally, similar metal chalcogenide clusters with identical stoichiometric compositions are chemically synthesized^20–25^. The optimized ground state geometry of Ti_6_Se_8_ is a distorted face-capped octahedron with 0 μ_B_ spin magnetic moment. Figure 1a shows the ground state optimized geometry of the Ti_6_Se_8_ cluster along with some bond lengths (in Å). The structure with 2 μ_B_ spin magnetic moment is ~0.14 eV higher in energy compared to the ground state geometry (see Supplementary Table 1). The HOMO-LUMO gap, adiabatic ionization energy (AIE), and adiabatic electron affinity (AEA) of the ground state (0 μ_B_) cluster are 0.44, 6.79, and 2.98 eV, respectively. Electron localization function (ELF) calculation (Fig. 1a) further reveals that the electrons are mostly localized on the Se atoms, which also explains the observed negative Hirshfeld^51^ charge (−0.15 |e|) on the selenium atoms. In contrast, the Ti atoms are seen to be positively charged (+0.26 | e |). The projected density of states (PDOS) and the molecular orbital (MO) diagram of the cluster is included in Fig. 1b. The PDOS diagram and the frontier orbital isosurfaces clearly show that both the HOMO and LUMO are composed of Ti-d orbitals, and Se has minor contributions.

**Fig. 2:**
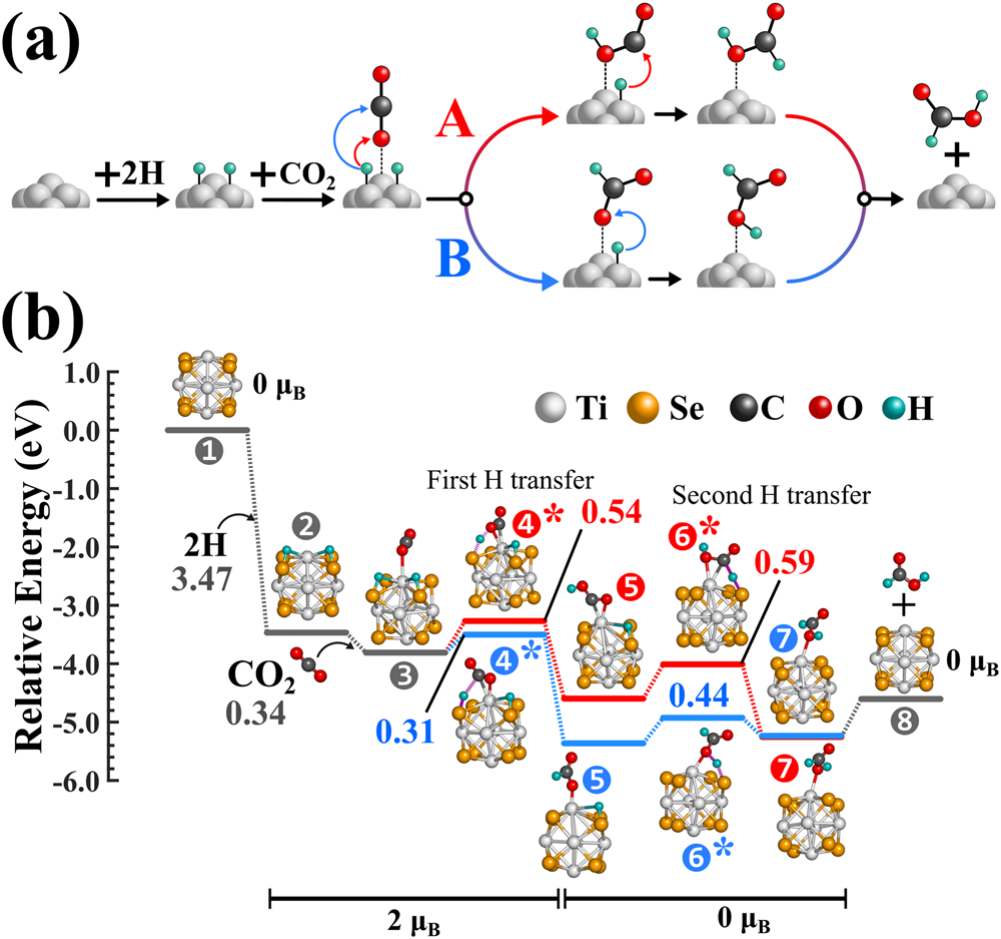
CO_2_ → HCOOH conversion pathways. **a** The schematic of CO_2_ → HCOOH conversion on a cluster surface showing two possible reaction pathways (A and B) and **b** the computed reaction pathways on the ground state Ti_6_Se_8_ cluster. The binding energies of H and CO_2_ are shown in grey, barrier heights of the two pathways (A and B) are shown in red and blue color, respectively. The transition states are marked with asterisk (*) symbol.

As a next step, we proceed to compute the favorable reaction pathway of CO_2_ hydrogenation on the Ti_6_Se_8_ cluster catalyst. Figure 2a shows a general schematic of the CO_2_ → HCOOH conversion on a cluster surface. For the present study, we have considered that the first step of the reaction is the adsorption of dissociated H atoms on the cluster surface, which is followed by the adsorption of a CO_2_ molecule at an adjacent site of the cluster. Our calculation (see Supplementary Fig. 1) reveals that the H_2_ dissociation barrier on the Ti_6_Se_8_ cluster is 1.08 eV (PBE/TZ2P); however, dissociated H atoms produced by any other experimental methods^52–55^ can also be used for the reaction as alternatives. Since the adsorption sites of CO_2_ and H atoms are different, the sequence by which they are adsorbed on the cluster surface is irrelevant to the present reaction. Further discussion regarding this and related arguments, specifically for the Ti_6_Se_8_ cluster, is given later on. Following the adsorption steps, we have found that the reaction has the possibility to proceed via two pathways depending on to which atom of the CO_2_ molecule (C or O) the first H atom gets transferred. As shown in Fig. 2a, we have designated these two pathways as (A) and (B) in red and blue color, respectively. In pathway A, the first H gets transferred to the nearest O atom of the CO_2_ molecule resulting in a COOH intermediate, and subsequent transfer of the 2nd H to the C atom of the intermediate yields the formic acid. In path B, the reaction proceeds via an HCOO intermediate since the first H gets attached to the C atom of CO_2_. The formic acid is then produced via the transfer of the second hydrogen from the cluster surface to the nearest O atom of the intermediate.

In Fig. 2b, we have shown both the calculated pathways on Ti_6_Se_8_ clusters. As we have shown in the schematics, the first step is considered to be the adsorption of the H atoms on the cluster surface. Although we expected that the H atoms would bind only to the Ti or Se site, upon adsorption, it was noticed that the dissociated H atoms favorably formed bridging bonds with the Ti and one of the nearby Se atoms with a net binding energy of 3.47 eV. This is evident by looking into the geometric structure of intermediate **2**, as shown in Fig. 2b. This is due to chalcogens like S or Se being able to form a much stronger bond with H compared to Ti. Wiberg bond indices (WBI) calculated by Natural bond orbital (NBO) analysis^56^ further revealed that the Se−H bond order is significantly higher (0.82-0.85) compared to the Ti−H bond order (0.07-0.09), showing that the Se−H interaction is the dominant one. Important to note that after H adsorption, the ground state spin-only magnetic moment is also altered to 2μ_B_. The bridging orientation of H and stronger Se−H interaction proves two things. First, even after the adsorption of H atoms, the Ti site remains free for CO_2_ adsorption. So there is minimal competition between H and CO_2_ adsorption on the cluster as long as the number of H atoms is reasonably low, and hence their adsorption order (i.e., which one among H or CO_2_ is adsorbed first) does not influence the calculated reaction pathway. Second, since the interaction of the dissociated H atoms is much stronger with Se than Ti, the presence of nearby H atoms does not affect the binding energy of the incoming CO_2_ at a lower H concentration. Therefore, it is expected that the reaction parameters will remain the same as long as the number of adsorbed H that are in close vicinity to CO_2_ remains low. Hence, although here we have considered the whole reaction pathway by coadsorbing both the hydrogen atoms, sequential adsorption, i.e., one H atom at a time, should not alter the reaction parameters. These observations and conclusions were also confirmed in previous investigations based on the Mo_6_S_8_ cluster^57,58^. It is noteworthy that the computed reaction pathways presented here are all based on the aforementioned assumptions, i.e., the number of adsorbed H atoms remains low. The influence of a higher number of adsorbed H on the reaction mechanism demands a separate study and is not the current focus of the paper. Following the H adsorption, the resulting cluster complex, i.e., intermediate **2** gets stabilized by 0.34 eV via the adsorption of CO_2_ on the Ti atom. This leads to structure **3**, as shown in Fig. 2b. We have observed that, in contrast to H, the CO_2_ molecule prefers to bind with the Ti site via one of the O atoms. Upon adsorption, the molecule adopts an angular orientation with respect to the cluster surface.

As shown previously, starting from **3**, the reaction can possibly proceed via two different pathways, namely A and B respectively. We have investigated both the pathways for Ti_6_Se_8_ clusters, and the barrier heights are included in Fig. 2b. From the diagram, it is seen that the two barrier heights of the first (4*) and second (6*) H transfer along pathway A (COOH intermediate, shown as red-colored 5 in Fig. 2b) are 0.54 and 0.59 eV, respectively. In contrast, the reaction pathway via the HCOO intermediate (i.e., pathway B) shows significantly lower barrier heights, 0.31 and 0.44 eV, respectively. The lowering of the second barrier height in pathway B can be attributed to the lowering of the intermediate (5) along with the transition states (6*), as shown in Fig. 2b. Due to the lower barriers, it is evident that the CO_2_ → HCOOH conversion on the Ti_6_Se_8_ clusters will preferably proceed via pathway B and through the HCOO intermediate (blue-colored 5 in Fig. 2b) despite the energies of the final products (7 in Fig. 2b) from both pathways are near identical. The sum of energies of the free cluster and formic acid are included in the same figure as 8 for reference purposes. It is also important to note that the ground state spin moments do not remain constant throughout the reaction. A second spin alteration is noticed after the first transition state. Thus, although 2μ_B_ spin magnetic moment is maintained till 4*, beginning from intermediate 5 in both A and B pathways, the ground state moment has altered to 0μ_B,_ which remained the same till the end of the reaction. The relative energies of all species in the reaction pathway considering the different magnetic moments are provided in the Supplementary Table 2 and 3.

### Effect of ligands on the reaction pathway of CO_2_ → HCOOH conversion

**Fig. 3:**
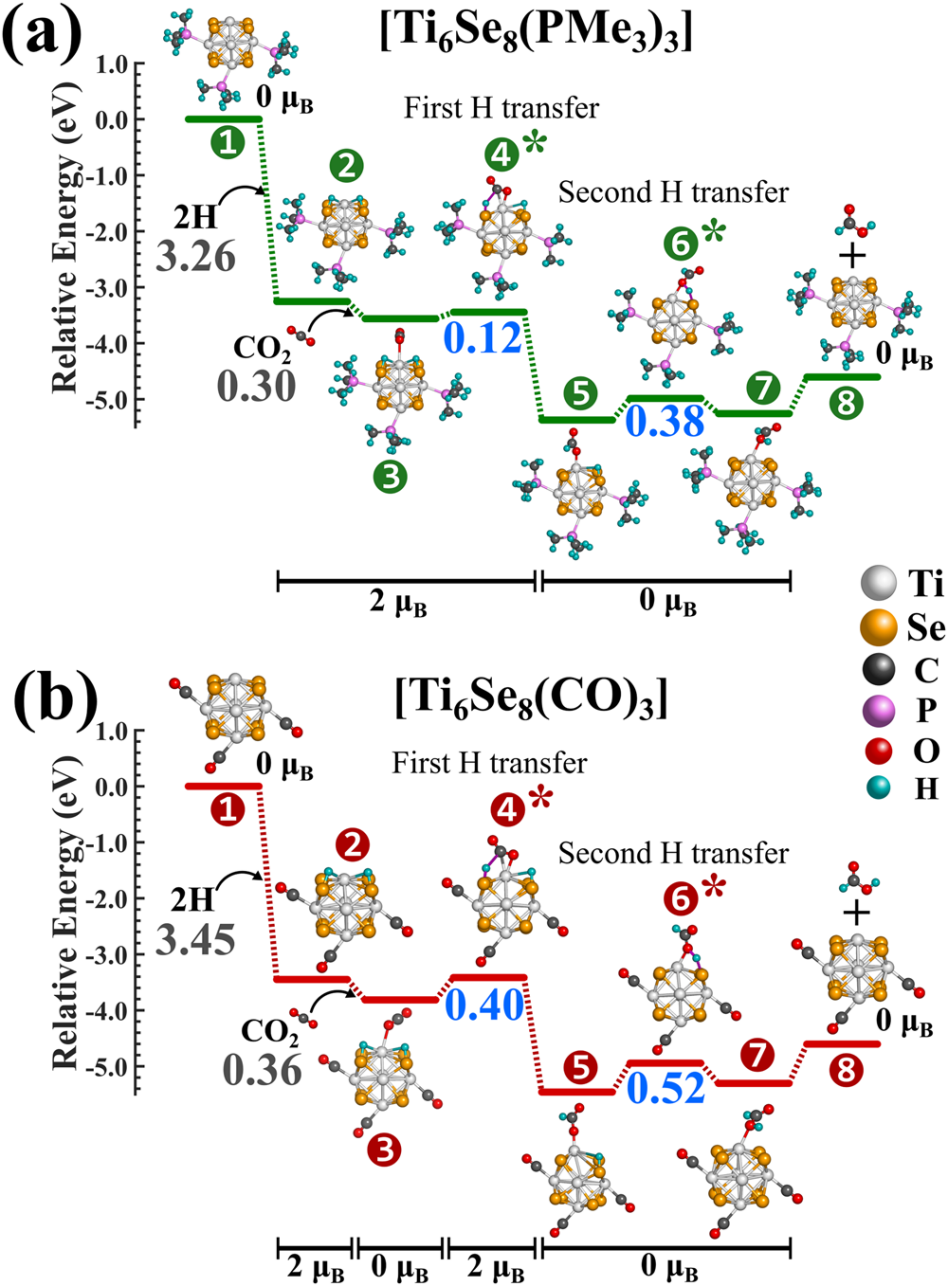
CO_2_ → HCOOH conversion on the Ti_6_Se_8_(PMe_3_)_3_ and Ti_6_Se_8_(CO)_3_ clusters. The most favorable CO_2_ → HCOOH conversion pathways on the (**a**) Ti_6_Se_8_(PMe_3_)_3_ and **b** Ti_6_Se_8_(CO)_3_ clusters. The binding energies of H and CO_2_ are shown in grey, barrier heights are shown in blue color. The transition states are marked with asterisk (*) symbol.

Following the calculation of the most favorable CO_2_ → HCOOH conversion route on the pristine Ti_6_Se_8_ cluster, we proceed to understand how ligands will influence the barrier heights of CO_2_ hydrogenation. To achieve this, we have chosen two different dative ligands, namely, PMe_3_ and CO. Based on the electronic effect, these two ligands differ widely from each other. The PMe_3_ ligand is a strong σ-donor but a poor π-acceptor. In contrast, the CO ligand is a strong electron donor as well as a good π-acceptor. Starting from the pristine Ti_6_Se_8_, we have sequentially increased the number of attached ligands with the Ti atoms of the cluster. In all cases, we have kept the reaction site unligated so that the reaction could proceed without any hindrance. In this present work, we have restricted ourselves to a maximum of three ligands of each type. The major reason behind this is that we have observed that going beyond three ligands (especially for PMe_3_) results in a major steric crowding in the vicinity of the reaction site. This results in a weakening of CO_2_/H binding and subsequent desorption from the cluster surface. Our test calculation on Ti_6_Se_8_(CO)_3_ cluster (see Supplementary Fig. 2) indicated that pathway B remains energetically favorable even after ligand attachment. Hence, for the ligated systems, only pathway B is computed and included in this study. Figures 3a, b show the reaction pathway of CO_2_ → HCOOH conversion on the Ti_6_Se_8_(PMe_3_)_3_ and Ti_6_Se_8_(CO)_3_ clusters, respectively. The results for the rest of the ligated clusters (i.e., Ti_6_Se_8_(PMe_3_)_n_ and Ti_6_Se_8_(CO)_n_, n = 1-2) are included in the Supplementary Table 4–6. As shown, the trend of the barrier heights for CO_2_ hydrogenation on the ligated clusters is observed to be very interesting. As reported, for all the CO-ligated clusters, including Ti_6_Se_8_(CO)_3_, both the CO_2_ hydrogenation barriers are shown to be higher compared to the Ti_6_Se_8_ cluster. It is interesting to note that the first barriers for all three Ti_6_Se_8_(CO)_n_ clusters are nearly the same ~0.40 eV. However, the second barrier is observed to increase by ~0.03-0.04 eV with the increment of the number of the attached CO ligands one at a time (see Supplementary Table 6). In contrast, an opposite trend is noticed for Ti_6_Se_8_(PMe_3_)_n_ clusters. All the PMe_3_ attached clusters show relatively lower barrier heights compared to Ti_6_Se_8_ for CO_2_ hydrogenation. In the case of PMe_3_ ligated clusters, both the hydrogenation barriers are observed to decrease with the increment of the number of ligands attached to the cluster. However, the first hydrogenation barrier falls off more rapidly compared to the second one. In the case of Ti_6_Se_8_(PMe_3_)_3_ cluster (Fig. 3a), the first hydrogenation barrier was calculated as 0.12 eV, which is a significant reduction compared to that of Ti_6_Se_8_ (i.e., 0.31 eV). The alteration of the second CO_2_ hydrogenation barrier (0.38 eV) was not as drastic as the first one, and in this case, a reduction of 0.06 eV was noticed compared to for Ti_6_Se_8_ cluster (0.44 eV). As shown in Fig. 3, upon analyzing the relative energies of all the chemical species, we have observed the overall reduction of the second hydrogenation barrier in the case of Ti_6_Se_8_(PMe_3_)_3_ cluster compared to Ti_6_Se_8_(CO)_3_ is due to the energetic lowering of the transition state (6*) as well as due to the energetic destabilization of the respective intermediate (5). Both of these factors simultaneously result in a lower second hydrogenation barrier for the Ti_6_Se_8_(PMe_3_)_3_ cluster. At this point, it is important to mention that we have observed that using a hybrid functional (e.g., PBE0) instead of PBE is altering the barrier heights marginally (see Supplementary Fig. 3). Therefore, it is expected that even with a different level of theory, the relative trends and the conclusions drawn herewith will still remain the same.

**Fig. 4:**
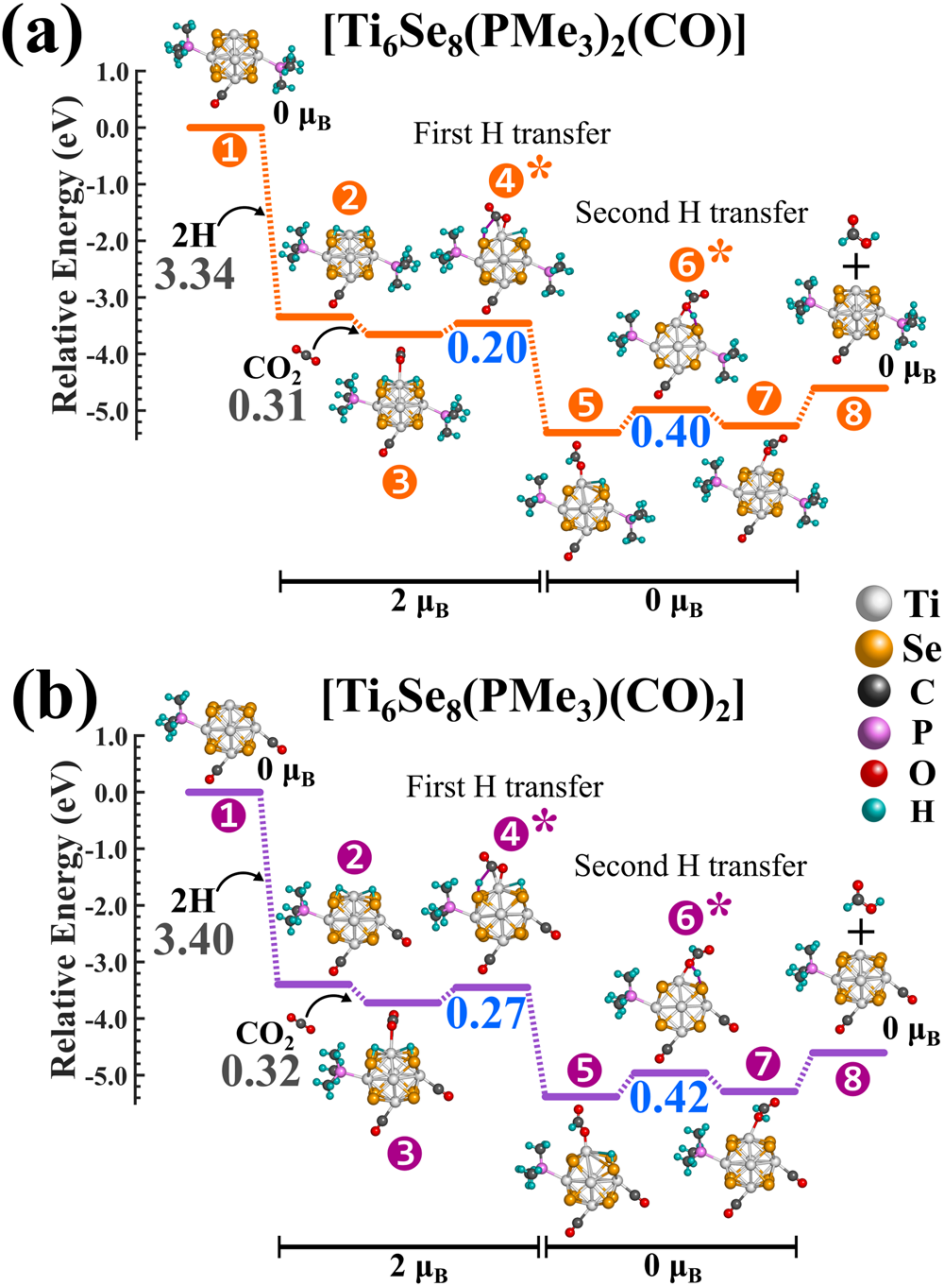
CO_2_ → HCOOH conversion on the [Ti_6_Se_8_(PMe_3_)_3-m_(CO)_m_] (m = 1,2) clusters. The most favorable CO_2_ → HCOOH conversion pathways on the (**a**) Ti_6_Se_8_(PMe_3_)_2_(CO) and **b** Ti_6_Se_8_(PMe_3_)(CO)_2_ clusters. The binding energies of H and CO_2_ are shown in grey, barrier heights are shown in blue color. The transition states are marked with asterisk (*) symbol.

**Fig. 5:**
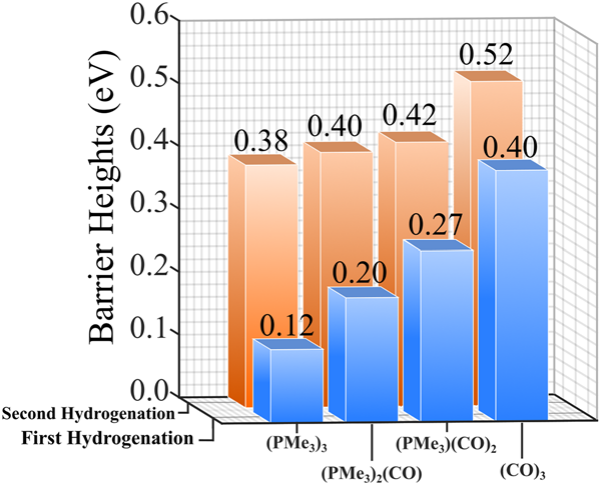
Ligand-induced barrier heights of CO_2_ → HCOOH conversion. The relative trend of the barrier heights for both the hydrogenation steps of [Ti_6_Se_8_(PMe_3_)_3-m_(CO)_m_] (m = 0-3) clusters. The plot shows smooth predictable alteration of barrier heights upon ligand substitution.

So far, we have shown that the barrier heights of CO_2_ hydrogenation on Ti_6_Se_8_ can effectively be decreased/increased by decorating the cluster with σ-donor (e.g., PMe_3_) or π-acceptor (e.g., CO) ligands. This provides a unique opportunity to predictably alter the reactivity of a cluster simply by changing the ratio of the attached ligands of both types. To further elaborate on this possibility, we started from the Ti_6_Se_8_(PMe_3_)_3_ cluster and sequentially replaced one PMe_3_ with one CO ligand at each step till we reached Ti_6_Se_8_(CO)_3_ cluster. The reaction profiles of these two intermediate clusters, i.e., [Ti_6_Se_8_(PMe_3_)_3-m_(CO)_m_] (m = 1,2) clusters along with all the optimized geometries are shown in Fig. 4. The relative energies of all species in the reaction pathway considering the different magnetic moments of [Ti_6_Se_8_(PMe_3_)_3-m_(CO)_m_] (m = 1,2) clusters are included the Supplementary Tables 7–8. As expected, both the barrier heights of these two clusters are observed to be intermediate in magnitude compared to the barrier heights obtained for the Ti_6_Se_8_(PMe_3_)_3_ and Ti_6_Se_8_(CO)_3_ clusters (Fig. 3). In Fig. 5, we have combined the barrier heights of both hydrogenation steps for all these four ligated clusters, i.e., [Ti_6_Se_8_(PMe_3_)_3-m_(CO)_m_] (m = 0-3), and as shown, it is now evident that by sequentially changing the ratio of the attached PMe_3_ and CO ligands, both the hydrogenation barriers can be altered in a controlled and stepwise manner. The range of alteration in the first and second barriers at each step is observed to be 0.07–0.13 and 0.02–0.10 eV, respectively. It is important to mention that by observing the stepwise reduction of barrier heights, one can argue that some of the barrier height differences between the adjacent step are not that significant considering the error range of the DFT methodology; however, it is impossible to ignore the observed systematic trend as the Fig. 5 show a near-smooth decrease of both the hydrogenation barriers as we sequentially increase the number of attached PMe_3_ ligands. This observation is crucial since, by using such strategies, it might be possible for experimentalists to predictably alter and control the rate of a particular reaction and hence the reactivity of the cluster. Moreover, by using a larger cluster with more available sites for ligand attachment and by using a stronger donor, the magnitude of stepwise barrier height reduction can further be increased. Additionally, it is noteworthy that although the stepwise reduction in barrier height for the present cluster may be low in some cases, the overall reduction of the hydrogenation barrier heights (0.14-0.28 eV, considering both hydrogenation steps) from Ti_6_Se_8_(CO)_3_ to Ti_6_Se_8_(PMe_3_)_3_ cluster is reasonably significant.

### Understanding the ligand-induced reactivity alteration of the Ti_6_Se_8_ cluster toward CO_2_ conversion

**Fig. 6:**
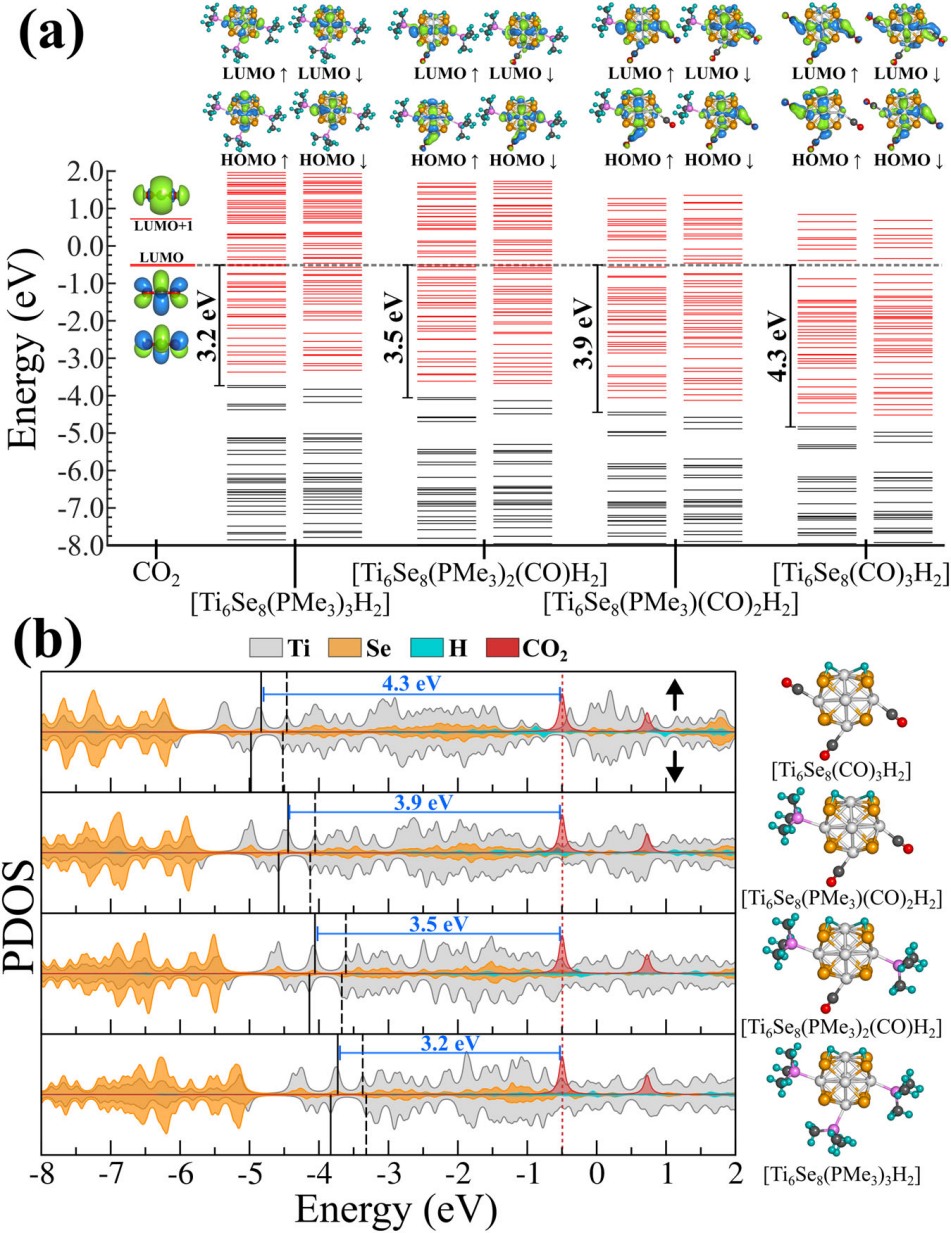
Ligand induced electronic property alteration of intermediate 2. **a** Molecular orbital (MO) and **b** projected density of states (PDOS) diagram of intermediate 2 for the [Ti_6_Se_8_(PMe_3_)_3-m_(CO)_m_] (m = 0-3) cluster. The black and red lines in **a** represents the occupied and unoccupied levels. In **b**, the location of HOMO and LUMO for each spin channel is marked by solid and dashed black lines respectively. The LUMO of CO_2_ molecule is indicated by a dashed red line.

**Fig. 7:**
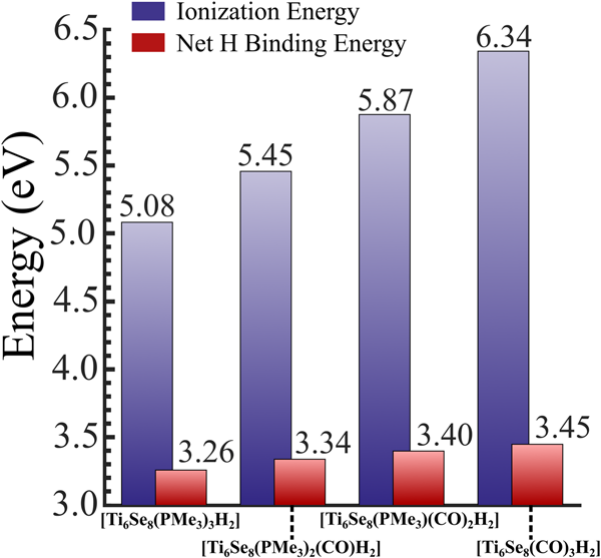
Ligand-induced alteration of AIE and net hydrogen binding energy of intermediate 2. The trend in the adiabatic ionization energy (AIE) and the net hydrogen binding energy of intermediate 2 for the [Ti_6_Se_8_(PMe_3_)_3-m_(CO)_m_] (m = 0-3) cluster. The plot shows a smooth variation of both properties upon ligand substitution.

We now consider the underlying electronic effects that are responsible for such alteration of the reactivity of the clusters. To achieve this, we have plotted the molecular orbital (MO) diagram and the projected density of states (PDOS) (Fig. 6) of intermediate 2 for the four ligated clusters. An alternative version of the same figure, along with the electronic structure of Ti_6_Se_8_ cluster, is included in the Supplementary Fig. 4. As shown earlier (Figs. 3 and 4), intermediate 2 is obtained via the adsorption of the H atoms on the cluster surface, and hence it can be considered one of the crucial species in the reaction pathway. From Fig. 6, we can see that increasing the number of PMe_3_ ligands at each step (right to left for **6a** and top to bottom for **6b**) creates an energetical upward shift of the whole electronic (MO) spectrum of intermediate 2. This shift is observed for both spin channels of intermediate 2 with minimal alteration in the HOMO-LUMO gaps of the same. It is noteworthy that the upward shift has also not perturbed the order of molecular orbitals or the occupation number of both spin channels (intermediate 2). In Fig. 6, we have also included the MO diagram and PDOS of a free CO_2_ molecule for reference purposes. The ligand-induced upward energetic shift of intermediate 2 results in the alteration of multiple important properties. First, the upward shift of the electronic (MO) spectrum results in the reduction of the energetic gap between the HOMO of intermediate 2 and LUMO of the free CO_2_ molecule, thereby favoring the orbital overlap and the charge transfer during the course of the reaction. As one can see from Fig. 6, the respective gap is considerably lower, i.e., 3.2 eV for [Ti_6_Se_8_(PMe_3_)_3_H_2_] cluster compared to 4.3 eV as obtained for [Ti_6_Se_8_(CO)_3_H_2_] cluster. Additionally, due to the ligand-induced upward shift of the electronic (MO) spectrum, intermediate 2 also becomes a better electron donor due to the reduction in the ionization energy. In Fig. 7, we have shown the trends in the ionization energy of intermediate 2 for all four clusters. As depicted, the adiabatic ionization energy (AIE) of the [Ti_6_Se_8_(PMe_3_)_3_H_2_] is observed to be significantly lower (5.08 eV) compared to the AIE of the [Ti_6_Se_8_(CO)_3_H_2_] cluster (6.34 eV) which further facilitates the charge transfer from the intermediate 2 to CO_2_. This argument can also be proven simply by looking at the accumulated charges on the CO_2_ molecule at the first transition state (i.e., 4* in Figs. 3 and 4). The Hirshfeld^51^ charge on CO_2_ at the respective transition state is more negative (-0.36 | e | ) in the Ti_6_Se_8_(PMe_3_)_3_ pathway compared to the Ti_6_Se_8_(CO)_3_ pathway (-0.26 | e | ). The rest of the two clusters show intermediate charges on CO_2_ and also follow the expected trend (see Supplementary Table 9). Apart from facilitating the charge transfer, it is also observed that the ligand-induced shift alters the binding energy of H atoms as well. Figure 7 also shows the net H binding energies (i.e., the binding energy of the H atom pair with the ligated cluster) for all four intermediates (2), and as shown, increasing the number of PMe_3_ ligands results in a reduction of the same. According to our calculation, the net H binding energy has reduced monotonically from 3.45 eV to 3.26 eV as we move from [Ti_6_Se_8_(CO)_3_H_2_] to [Ti_6_Se_8_(PMe_3_)_3_H_2_] cluster. One possible explanation for such reduction is that as the electronic spectrum shifts upward, the bonding orbitals of the attached hydrogens get energetically destabilized, thereby facilitating their release from the cluster surface. In other words, the upward shifting of the electronic (MO) spectrum along with the H bonding molecular orbitals results in the weakening of the bond and, thereby, the binding energy with the cluster. To summarize, we can conclude that the ligand-induced upward shifting of the electronic spectrum assists the H release from the cluster surface and also expedites the charge transfer from the cluster to CO_2_ by improving the orbital overlap and reducing the ionization energy. Since the overall ligand-induced alteration of the H binding energy is smaller compared to the AIE, we think that the facilitation of the charge transfer due to the donor ligand attachment plays a dominant role in the observed barrier height reduction. Therefore, controlling the degree of the shift via ligands, as shown in this article, provides a way to predictably alter the barrier heights and hence the reactivity of the cluster.

## Discussion

The present study provides valuable insight into how the rate of CO_2_ conversion to formic acid can be controlled by attaching suitable combinations of donor/acceptor ligands to the Ti_6_Se_8_ cluster catalyst. Our investigation reveals that attaching σ-donor (e.g., PMe_3_)/ π-acceptor (e.g., CO) ligands to the cluster induces an upward/downward shift of the electronic spectrum of the cluster without altering the sequence or the occupancy of the electronic levels. We have further shown that such ligand-induced alteration of the electronic levels also results in a change in the reactivity of the cluster in a predictable manner. This provides a unique opportunity to alter the barrier heights of the CO_2_ conversion in a progressive and predictable manner simply by attaching different combinations of donor/acceptor ligands to the cluster. Analysis of our computational results reveals that the alteration of the barrier is due to two different electronic effects. First, the upward shift of the electronic levels induces by the donor ligands lowers the ionization energy of the cluster and thereby facilitates the charge transfer toward CO_2_. Second, the same shift also weakens the binding of H atoms to the cluster and hence assists the H-transfer process. While the present studies are based on Ti_6_Se_8_ clusters, the insight gained into the various mechanisms controlling the barrier should be applicable to other metal chalcogenide clusters as well. It is noteworthy that the influence of other donor/acceptor ligands and the effect of various solvents on the barrier heights remain as open questions that demand separate in-depth studies. Despite the fact, as mentioned earlier, using synthetic chemical techniques, it is now possible to synthesize the ligated metal chalcogenide clusters in solutions. Therefore, we hope the present study will motivate experimental investigation into the facile synthesis of formic acid, thereby reducing the environmental CO_2_ concentration and converting it into useful products using metal chalcogenide clusters.

## Methods

### Computational details

All the reported results in this paper are calculated by using the Amsterdam Density Functional (ADF) package^59^. The gradient-corrected Perdew, Burke, and Ernzerhof (GGA-PBE) exchange-correlation functional is used for all computations^60^. The Slater-type triple ζ basis sets with two polarization functions (TZ2P) and large frozen electron cores are used for all the elements^61,62^. The frozen core orbitals are expressed in an auxiliary set of Slater-type basis functions that is different from the valence set. The scalar relativistic corrections are incorporated via the zero-order regular approximation (ZORA)^63,64^. The Hessian-based quasi-Newton method, without any symmetry constraints, is utilized for all optimizations^65^. To check the validity of the optimized structures, analytical frequency calculations^66,67^ are performed, and it is ensured that all the normal modes of vibrations for the minima structures are real and positive, whereas all the transition states are first-order saddle points having only one imaginary frequency with significant magnitude. To verify that the respective transition states are connected to the reported minima at the left and the right-hand side of the transition state, the intrinsic reaction coordinate (IRC) calculations^68^ are also performed. Since the geometry of all the transition states remained similar even after ligand attachment, the IRC calculations are performed only for the pristine cluster. This is also due to the high computational costs associated with each IRC calculation. The dispersion correction is included via Grimme’s DFT-D3 method with the Becke−Johnson damping^69^. The natural bond orbital (NBO) calculations are performed with NBO 6.0 program^56^ as implemented within ADF^59^ package. It is also confirmed that the spin contamination errors associated with all the open shell systems are negligible and within a reasonable level. A wide range of spin multiplicities are investigated during all optimizations, including for all the species along the reaction pathways, and only the ground state structures were selected for all cases.

## Supplementary information

Supplementary Information

## Data Availability

The optimized cartesian coordinates of the transition state structures, relative energies, barrier heights, and Hirshfeld charges are given in the Supplementary Information. Any additional data reported herewith are available from the corresponding authors upon reasonable request.
