# Supplementary material for: Converting CO2 to formic acid by tuning quantum states in metal chalcogenide clusters
Source: Commun Chem. 2023 Mar 21;6:53. doi: 10.1038/s42004-023-00851-3 (PMC10027883; doi:10.1038/s42004-023-00851-3)

Supplementary Information

Converting CO_2 to Formic Acid by Tuning Quantum States in Metal Chalcogenide Clusters

Turbasu Sengupta* and Shiv N. Khanna*.

Department of Physics, Virginia Commonwealth University, Richmond, VA, 23284-2000.

***Corresponding Authors:** senguptat@vcu.edu, snkhanna@vcu.edu

Index

		P. No
**Supplementary Table 1**	Relative energies of Ti_6Se_8 clusters with different magnetic moments	S3
**Supplementary Table 2**	Relative energies of each species with different magnetic moments in the CO_2→HCOOH pathway on Ti_6Se_8 clusters	S3
**Supplementary Table 3**	Relative energies of each species with respect to 1, considering the lowest energy spin state of each species for the CO_2→HCOOH pathway on Ti_6Se_8 clusters	S3
**Supplementary Table 4**	Relative energies of each species with different magnetic moments in the CO_2→HCOOH pathway on $Ti_6Se_8(PMe_3)_n$ [n=1-3] clusters	S4
**Supplementary Table 5**	Relative energies of each species with different magnetic moments in the CO_2→HCOOH pathway on $Ti_6Se_8(CO)_n$ [n=1-3] clusters	S5
**Supplementary Table 6**	Barrier heights of CO_2→HCOOH pathway on the Ti_6Se_8, $Ti_6Se_8(PMe_3)_n$ and $Ti_6Se_8(CO)_n$, [n=1-3] clusters and the CO_2 binding energies with intermediate 2	S5
**Supplementary Table 7**	Relative energies of each species with different magnetic moments in the CO_2→HCOOH pathway on $[Ti_6Se_8(PMe_3)_{3-m}(CO)_m]$ (m=1,2) clusters	S6
**Supplementary Table 8**	Relative energies of each species with respect to 1, considering the lowest energy spin state of each species for the CO_2→HCOOH pathway on $[Ti_6Se_8(PMe_3)_{3-m}(CO)_m]$ (m=0, 3) clusters	S6
**Supplementary Table 9**	Hirshfeld charges on CO_2 at the first transition state (4*) in the CO_2→HCOOH pathway on $[Ti_6Se_8(PMe_3)_{3-m}(CO)_m]$ (m=0-3) clusters	S6
**Supplementary Table 10**	Optimized (PBE/TZ2P) transition state (4* and 6*) coordinates for the reaction pathways	S7-S11
**Supplementary Figure 1**	Dissociation Pathway of H_2 on pristine Ti_6Se_8 cluster (GGA-PBE/TZ2P)	S12
**Supplementary Figure 2**	The schematic of CO_2→HCOOH conversion on the $Ti_6Se_8(CO)_3$ cluster surface showing two possible reaction pathways (A and B)	S13
**Supplementary Figure 3**	Calculated reaction pathways of CO_2→HCOOH conversion on the (a) Ti_6Se_8 and (b)$Ti_6Se_8(CO)_3$ cluster in PBE0/TZ2P level of theory	S14
**Supplementary Figure 4**	(a) Molecular orbital and (b) projected density of states diagram of intermediate 2 for the $[Ti_6Se_8(PMe_3)_{3-m}(CO)_m]$, (m=0-3) and Ti_6Se_8 cluster.	S15

Supplementary Table 1. Relative energies of Ti_6Se_8 clusters with different magnetic moments (ground state is highlighted with bold font)

Ti_6Se_8

Mag. Moment (μ_B)	Energy (eV)	Relative Energy (eV)
**0**	**-77.54871079**	**0.0000**
2	-77.41181496	0.1369
4	-77.19944526	0.3493
6	-76.74774475	0.8010
8	-75.93669342	1.6120
10	-74.81262042	2.7361
12	-73.38786278	4.1608
14	-71.65961688	5.8891

Supplementary Table 2. Relative energies of each species with different magnetic moments in the CO_2→HCOOH pathway on Ti_6Se_8 clusters (ground state is highlighted with bold font)

Mag. Moment (μ_B)	Relative Energy (eV)						
	**1**	**2**	**3**	**4***	**5**	**6***	**7**
**Pathway A**							
0	**0.0000**	0.0303	0.0158	0.0272	**0.0000**	**0.0000**	**0.0000**
2	0.1369	**0.0000**	**0.0000**	**0.0000**	0.0651	0.1032	0.1459
4	0.3493	0.0622	0.0347	0.2268	0.0166	0.3112	0.2607
	**1**	**2**	**3**	**4***	**5**	**6***	**7**
**Pathway B**							
0	Same as Pathway-a	Same as Pathway-a	Same as Pathway-a	0.0780	**0.0000**	**0.0000**	**0.0000**
2				**0.0000**	0.0151	0.1017	0.1451
4				0.1416	0.2992	0.3081	0.3097

Supplementary Table 3. Relative energies of each species with respect to 1, considering the lowest energy spin state of each species for the CO_2→HCOOH pathway on Ti_6Se_8 clusters

Relative Energy (eV)							
**1**	**2**	**3**	**4***	**5**	**6***	**7**	**8**
**Pathway A**							
0	-3.46778403	-3.8104683	-3.26893593	-4.59754438	-4.01092137	-5.25459133	-4.60396027
**1**	**2**	**3**	**4***	**5**	**6***	**7**	**8**
**Pathway B**							
0	-3.46778403	-3.8104683	-3.50158706	-5.36332557	-4.92671042	-5.25265284	-4.60396027

Supplementary Table 4. Relative energies of each species with different magnetic moments in the $CO_2 \rightarrow HCOOH$ pathway on $Ti_6Se_8(PMe_3)_n$ [n=1-3] clusters (ground state is highlighted with bold font)

Mag. Moment (μ_B)	Relative Energy (eV)						
	**1**	**2**	**3**	**4***	**5**	**6***	**7**
**n=1**							
0	**0.0000**	0.0660	0.0618	0.0850	**0.0000**	**0.0000**	**0.0000**
2	0.1131	**0.0000**	**0.0000**	**0.0000**	0.0309	0.1236	0.1533
4	0.2530	0.0869	0.0779	0.1523	0.2584	0.2754	0.2644
	**1**	**2**	**3**	**4***	**5**	**6***	**7**
**n=2**							
0	**0.0000**	0.0295	0.0274	0.1308	**0.0000**	**0.0000**	**0.0000**
2	0.1675	**0.0000**	**0.0000**	**0.0000**	0.0209	0.1551	0.1777
4	0.2188	0.0494	0.0436	0.0474	0.1572	0.2621	0.2285
	**1**	**2**	**3**	**4***	**5**	**6***	**7**
**n=3**							
0	**0.0000**	0.0803	0.0659	0.1356	**0.0000**	**0.0000**	**0.0000**
2	0.1520	**0.0000**	**0.0000**	**0.0000**	0.0414	0.1542	0.1514
4	0.1949	0.0723	0.0477	0.0807	0.2088	0.1701	0.1969

Supplementary Table 5. Relative energies of each species with different magnetic moments in the $CO_2 \rightarrow HCOOH$ pathway on $Ti_6Se_8(CO)_n$ [n=1-3] clusters (ground state is highlighted with bold font)

Mag. Moment (μ_B)	Relative Energy (eV)						
	**1**	**2**	**3**	**4***	**5**	**6***	**7**
**n=1**							
0	**0.0000**	**0.0000**	0.0267	0.1881	**0.0000**	**0.0000**	**0.0000**
2	0.1264	0.0021	**0.0000**	**0.0000**	0.0371	0.0976	0.1421
4	0.2718	0.0584	0.0794	0.1404	--	0.2553	0.2647
	**1**	**2**	**3**	**4***	**5**	**6***	**7**
**n=2**							
0	**0.0000**	0.0112	0.0229	0.1318	**0.0000**	**0.0000**	**0.0000**
2	0.1402	**0.0000**	**0.0000**	**0.0000**	0.0663	0.1185	0.1584
4	0.2556	0.0978	0.0958	0.2094	0.2771	0.3524	0.2358
	**1**	**2**	**3**	**4***	**5**	**6***	**7**
**n=3**							
0	**0.0000**	0.0010	**0.0000**	0.0857	**0.0000**	**0.0000**	**0.0000**
2	0.1387	**0.0000**	0.0136	**0.0000**	0.1577	0.1319	0.1586
4	0.2552	0.1346	0.1350	0.2040	0.3424	0.2491	0.2278

Supplementary Table 6. Barrier heights of $CO_2 \rightarrow HCOOH$ pathway on the Ti_6Se_8, $Ti_6Se_8(PMe_3)_n$ and $Ti_6Se_8(CO)_n$, [n=1-3] clusters and the CO_2 binding energies with intermediate 2

Cluster	First Barrier (eV)	Second Barrier (eV)	CO_2 BE (eV)
**Ti_6Se_8**	0.31	0.44	0.343
**$Ti_6Se_8(CO)_n$ (n=1 to 3) Series**			
**$Ti_6Se_8(CO)$**	0.40	0.45	0.385
**$Ti_6Se_8(CO)_2$**	0.40	0.49	0.367
**$Ti_6Se_8(CO)_3$**	0.40	0.52	0.365
**$Ti_6Se_8(PMe_3)_n$ (n=1 to 3) Series**			
**$Ti_6Se_8(PMe_3)$**	0.26	0.45	0.336
**$Ti_6Se_8(PMe_3)_2$**	0.20	0.42	0.322
**$Ti_6Se_8(PMe_3)_3$**	0.12	0.38	0.307

Supplementary Table 7. Relative energies of each species with different magnetic moments in the CO_2→HCOOH pathway on [$Ti_6Se_8(PMe_3)_{3-m}(CO)_m$] (m=1,2) clusters (ground state is highlighted with bold font)

Mag. Moment (μ_B)	Relative Energy (eV)						
	**1**	**2**	**3**	**4***	**5**	**6***	**7**
**$Ti_6Se_8(PMe_3)_2(CO)$**							
0	**0.0000**	0.0450	0.0645	0.0784	**0.0000**	**0.0000**	**0.0000**
2	0.1369	**0.0000**	**0.0000**	**0.0000**	0.0784	0.1707	0.1601
4	0.2431	0.1287	0.1016	0.1502	0.1924	0.3181	0.2495
	**1**	**2**	**3**	**4***	**5**	**6***	**7**
**$Ti_6Se_8(PMe_3)(CO)_2$**							
0	**0.0000**	0.0144	0.0010	0.0755	**0.0000**	**0.0000**	**0.0000**
2	0.1588	**0.0000**	**0.0000**	**0.0000**	0.0762	0.1613	0.1811
4	0.2676	0.1470	0.1320	0.1758	0.2735	0.3097	0.2504

Supplementary Table 8. Relative energies of each species with respect to 1, considering the lowest energy spin state of each species for the CO_2→HCOOH pathway on [$Ti_6Se_8(PMe_3)_{3-m}(CO)_m$] (m=0, 3) clusters

Relative Energy (eV)							
**1**	**2**	**3**	**4***	**5**	**6***	**7**	**8**
**$Ti_6Se_8(PMe_3)_3$**							
0	-3.25539683	-3.56270158	-3.44547347	-5.37165125	-4.9853143	-5.2571158	-4.60396027
**$Ti_6Se_8(PMe_3)_2(CO)$**							
0	-3.34490074	-3.6556447	-3.45669179	-5.39030767	-4.98470202	-5.26896327	-4.60396027
**$Ti_6Se_8(PMe_3)(CO)_2$**							
0	-3.39536777	-3.7211709	-3.44846738	-5.38075265	-4.95989944	-5.28746165	-4.60396027
**$Ti_6Se_8(CO)_3$**							
0	-3.4495557	-3.81472764	-3.41530261	-5.46041891	-4.94349536	-5.30420742	-4.60396027

Supplementary Table 9. Hirshfeld charges on CO_2 at the first transition state (4*) in the CO_2→HCOOH pathway on [$Ti_6Se_8(PMe_3)_{3-m}(CO)_m$] (m=0-3) clusters

Cluster	Hirshfeld Charge on CO_2 (|e|)
**[$Ti_6Se_8(PMe_3)_3$]**	-0.36
**[$Ti_6Se_8(PMe_3)_2(CO)$]**	-0.33
**[$Ti_6Se_8(PMe_3)(CO)_2$]**	-0.30
**[$Ti_6Se_8(CO)_3$]**	-0.26

Supplementary Table 10. Optimized (PBE/TZ2P) transition state (4* and 6*) coordinates for the reaction pathways

Ti_6Se_8							
**Pathway-A**							
**4***				**6***			
Ti	-2.937832	1.782595	1.796321	Ti	-0.882249	15.283378	-0.478328
Ti	-1.230068	-0.460096	1.881964	Ti	0.958493	12.948145	-0.578052
Ti	-2.617186	-0.498117	4.164977	Ti	-0.547491	12.933675	1.737743
Ti	-2.895673	-0.796359	-0.450247	Ti	-0.704401	12.806880	-2.850599
Ti	-4.181640	-0.746657	1.864403	Ti	-1.982895	12.678456	-0.506066
Ti	-2.506800	-2.884997	1.988883	Ti	-0.387434	10.556964	-0.495915
Se	-4.514153	1.074310	-0.056237	Se	-2.439000	14.507716	-2.382701
Se	-0.898332	1.043137	-0.296509	Se	1.117204	14.673247	-2.563538
Se	-0.919853	1.474339	3.641970	Se	1.075548	14.905276	1.061883
Se	-4.531345	1.103811	3.649619	Se	-2.494867	14.504914	1.313581
Se	-4.351016	-2.736523	0.164871	Se	-2.156660	10.778403	-2.296797
Se	-4.213716	-2.505346	3.803063	Se	-2.053455	10.892849	1.388137
Se	-0.691197	-2.115092	3.717898	Se	1.432528	11.302722	1.258653
Se	-0.829498	-2.236651	0.096303	Se	1.335625	11.242263	-2.380689
C	-3.710827	3.687248	2.203769	C	-0.814321	17.214474	-1.620639
O	-4.500777	4.572728	2.321791	O	-0.831078	18.048423	-2.473466
O	-2.335620	3.733925	2.273046	O	-1.417388	17.420647	-0.320957
H	-1.558629	2.405940	-0.160411	H	0.239895	15.976754	-2.070229
H	-1.686356	2.918156	3.087718	H	-2.085745	18.141638	-0.319995
**Pathway-B**							
**4***				**6***			
Ti	-4.534438	4.728492	1.339932	Ti	12.007050	0.067615	2.538919
Ti	-2.871900	2.471903	1.377199	Ti	13.769226	-2.193159	2.229627
Ti	-4.335553	2.329978	3.769324	Ti	12.326210	-2.446051	4.565584
Ti	-4.482162	2.088115	-0.867590	Ti	12.190229	-2.210539	-0.034587
Ti	-5.748464	2.101907	1.482515	Ti	10.817854	-2.481202	2.270623
Ti	-4.043457	-0.038685	1.551204	Ti	12.485630	-4.611818	2.161989
Se	-6.150956	3.915526	-0.429350	Se	10.434973	-0.537396	0.560655
Se	-2.493668	3.912767	-0.836366	Se	14.030061	-0.405170	0.347396
Se	-2.561550	4.269126	3.342069	Se	13.914436	-0.439036	4.114914
Se	-6.111419	3.996344	3.223593	Se	10.362874	-0.748052	4.188328
Se	-5.902353	0.118300	-0.219981	Se	10.660476	-4.228286	0.391008
Se	-5.768048	0.274128	3.388517	Se	10.726699	-4.362394	4.039756
Se	-2.277800	0.859894	3.245804	Se	14.242639	-4.014172	3.979539
Se	-2.412197	0.632668	-0.341527	Se	14.162383	-3.882813	0.364683
C	-4.638489	6.806426	2.017869	C	11.924626	2.848011	0.983478
O	-5.018342	7.707775	2.710401	O	12.293253	3.256488	-0.085654
O	-3.989779	6.642056	0.917982	O	12.533447	1.842616	1.665792
H	-3.174880	5.255416	-0.788330	H	13.314053	1.046701	0.883627
H	-3.615924	5.426328	3.133580	H	11.060283	3.255336	1.548021

Ligated Clusters							
[$Ti_6Se_8(PMe_3)_3$]							
4*				6*			
Ti	-7.805796	12.405217	33.844382	Ti	-7.400008	9.298238	2.056083
Ti	-6.297257	10.119198	33.760520	Ti	-5.628430	7.140625	1.556657
Ti	-7.746213	9.969849	36.235015	Ti	-6.988295	6.616294	3.910134
Ti	-7.863980	9.784987	31.486693	Ti	-7.247927	7.257878	-0.730516
Ti	-9.268199	9.846756	33.961495	Ti	-8.603438	6.842134	1.653627
Ti	-7.618226	7.660667	33.897283	Ti	-6.915697	4.679595	1.275531
Se	-9.447669	11.639728	31.987895	Se	-9.026617	8.806359	-0.016277
Se	-5.799506	11.463106	31.509172	Se	-5.383564	8.977614	-0.319754
Se	-5.855150	11.865939	35.716343	Se	-5.430191	8.807908	3.486763
Se	-9.516139	11.778272	35.617282	Se	-9.030288	8.447529	3.655867
Se	-9.493172	7.908001	32.217113	Se	-8.811361	5.151314	-0.295202
Se	-9.349779	8.079076	35.879843	Se	-8.665430	4.873322	3.338533
Se	-5.799255	8.479077	35.681940	Se	-5.074847	5.214452	3.192768
Se	-5.867916	8.187875	32.110969	Se	-5.232102	5.480073	-0.382651
H	-6.385481	12.845331	31.528482	H	-6.144889	10.359406	0.298303
H	-6.758203	13.141483	35.536914	H	-8.609004	12.257608	1.093372
C	-7.909194	14.484697	34.252464	C	-7.689592	12.024978	0.512469
O	-7.186616	14.235023	33.204822	O	-6.919833	11.111311	1.136597
O	-8.278586	15.471768	34.838499	O	-7.415880	12.549866	-0.539498
P	-8.017928	11.108856	38.552263	P	-6.963241	7.476362	6.351233
P	-7.273236	5.316568	34.947827	P	-6.762220	2.250123	2.147466
P	-7.720403	8.522639	29.241100	P	-7.065471	6.362621	-3.153493
C	-6.845617	10.508083	39.839296	C	-5.413800	7.001668	7.222806
C	-7.852624	12.930632	38.675575	C	-7.122794	9.257472	6.747057
C	-9.662330	10.783802	39.305188	C	-8.278385	6.705238	7.380929
C	-7.091418	5.170422	36.765898	C	-6.529278	1.893255	3.929507
C	-8.671226	4.176607	34.583393	C	-8.258797	1.267595	1.725626
C	-5.810666	4.402096	34.308075	C	-5.401313	1.290784	1.361570
C	-6.286019	8.992514	28.192217	C	-5.547617	6.947946	-4.008026
C	-7.646205	6.691253	29.240943	C	-7.059983	4.561035	-3.489373
C	-9.166646	8.871356	28.156537	C	-8.424342	6.976334	-4.232603
H	-6.945327	9.420186	39.943324	H	-5.267502	5.917401	7.138050
H	-5.820522	10.731323	39.517731	H	-4.571430	7.499598	6.726255
H	-7.037929	10.986824	40.809309	H	-5.443892	7.288992	8.282893
H	-6.824707	13.214769	38.420467	H	-6.314998	9.804697	6.247217
H	-8.532464	13.402396	37.955507	H	-8.081108	9.623464	6.360670
H	-8.087902	13.270178	39.693224	H	-7.069131	9.412672	7.833091
H	-10.431609	11.212387	38.650716	H	-9.254220	7.007883	6.980690
H	-9.825371	9.700466	39.364400	H	-8.202366	5.613101	7.306106
H	-9.735898	11.227752	40.307298	H	-8.196824	7.009954	8.433465
H	-6.201707	5.730661	37.076744	H	-5.579298	2.331300	4.256974
H	-7.969430	5.624477	37.240869	H	-7.343663	2.366398	4.490799

H	-7.000351	4.117323	37.064744	H	-6.527426	0.809089	4.105677
H	-9.585104	4.599657	35.019140	H	-9.117859	1.708162	2.246927
H	-8.810603	4.108174	33.497016	H	-8.443672	1.334204	0.646051
H	-8.491509	3.174069	34.995680	H	-8.143876	0.214456	2.017293
H	-5.879616	4.330295	33.215301	H	-5.511047	1.334045	0.270537
H	-4.906535	4.972073	34.556360	H	-4.444749	1.757536	1.628102
H	-5.745069	3.394100	34.740202	H	-5.406284	0.241758	1.688824
H	-6.291872	10.078363	28.036116	H	-5.495763	8.041958	-3.944182
H	-5.365065	8.728444	28.726974	H	-4.677305	6.533744	-3.483742
H	-6.314412	8.478847	27.221444	H	-5.535066	6.635039	-5.061083
H	-6.750433	6.377652	29.789992	H	-6.233099	4.103575	-2.933330
H	-8.529556	6.303362	29.761939	H	-8.002356	4.130420	-3.131142
H	-7.613697	6.306556	28.212677	H	-6.942448	4.371868	-4.565006
H	-10.076592	8.531445	28.667065	H	-9.378040	6.594068	-3.847333
H	-9.247598	9.953826	27.994437	H	-8.452182	8.072564	-4.191416
H	-9.074532	8.362911	27.186916	H	-8.288867	6.651600	-5.273599
**[$Ti_6Se_8(PMe_3)_2(CO)$]**							
**4***				**6***			
Ti	-0.003759	2.352787	-0.114193	Ti	-1.624029	10.004137	-0.947592
Ti	1.458288	0.025426	-0.232808	Ti	0.153744	7.819249	-1.491118
Ti	0.087760	-0.077140	2.294448	Ti	-1.202551	7.340134	0.898599
Ti	-0.192738	-0.272372	-2.480350	Ti	-1.472796	8.008808	-3.761564
Ti	-1.503800	-0.173067	0.039567	Ti	-2.845885	7.522552	-1.387303
Ti	0.125826	-2.431419	-0.075688	Ti	-1.141395	5.376234	-1.776079
Se	-1.715372	1.621199	-1.919940	Se	-3.258394	9.533546	-3.003150
Se	1.901129	1.373921	-2.496521	Se	0.373609	9.722411	-3.308401
Se	1.976911	1.791201	1.699674	Se	0.347112	9.492378	0.463860
Se	-1.675016	1.753521	1.701303	Se	-3.260332	9.156558	0.631501
Se	-1.811869	-2.098843	-1.686388	Se	-3.054977	5.890615	-3.331077
Se	-1.546682	-1.931414	1.950674	Se	-2.878134	5.596207	0.308258
Se	1.995035	-1.583433	1.675966	Se	0.691866	5.924504	0.154911
Se	1.805237	-1.885708	-1.913988	Se	0.527229	6.223230	-3.446994
H	1.326742	2.761350	-2.453922	H	-0.380076	11.098285	-2.663864
H	1.062863	3.071608	1.546142	H	-2.791926	13.054127	-1.816910
C	-0.027573	4.439202	0.334911	C	-1.899333	12.781234	-2.420322
O	0.635802	4.181141	-0.746229	O	-1.154014	11.834135	-1.810212
O	-0.350946	5.420331	0.953955	O	-1.624951	13.289456	-3.479138
P	-0.134286	1.071750	4.615239	P	-1.305116	8.165388	3.347644
P	-0.084995	-1.540043	-4.727885	P	-1.390786	7.071893	-6.166465
C	1.043153	0.444708	5.883122	C	0.081456	7.484124	4.346221
C	0.061649	2.889851	4.734891	C	-1.268109	9.953153	3.740870
C	-1.774954	0.763865	5.381294	C	-2.801038	7.569438	4.232210
C	-1.495370	-1.111836	-5.829820	C	0.040785	7.738054	-7.106636
C	1.388742	-1.144582	-5.750181	C	-1.306123	5.271757	-6.493290

C	-0.112670	-3.371030	-4.717116	C	-2.843934	7.605037	-7.160243
H	0.928219	-0.642190	5.981707	H	0.082341	6.389742	4.265342
H	2.068615	0.655413	5.554531	H	1.028006	7.859109	3.937294
H	0.866361	0.919808	6.857791	H	-0.006235	7.773794	5.402450
H	1.089676	3.157703	4.462956	H	-0.328577	10.380421	3.371770
H	-0.623025	3.373678	4.027635	H	-2.100638	10.446219	3.225125
H	-0.150527	3.232210	5.756616	H	-1.352544	10.107207	4.825059
H	-2.547269	1.204572	4.738716	H	-3.683683	8.017701	3.759121
H	-1.949992	-0.317600	5.440613	H	-2.873568	6.479342	4.130483
H	-1.830410	1.204821	6.385803	H	-2.769362	7.841202	5.296143
H	-2.430352	-1.400614	-5.333157	H	0.030896	8.833901	-7.054941
H	-1.513707	-0.027113	-5.995331	H	0.962508	7.380801	-6.630538
H	-1.418467	-1.626778	-6.797257	H	0.014077	7.416107	-8.156689
H	1.445473	-0.059462	-5.901342	H	-0.413581	4.863782	-6.005043
H	2.285006	-1.462755	-5.203301	H	-2.191017	4.789375	-6.061999
H	1.345245	-1.652368	-6.723262	H	-1.263719	5.084280	-7.574667
H	0.746363	-3.733617	-4.140375	H	-3.748673	7.171644	-6.715787
H	-1.031477	-3.708342	-4.223065	H	-2.930541	8.698288	-7.121559
H	-0.070248	-3.761933	-5.742568	H	-2.753517	7.281159	-8.206251
C	0.491545	-4.094231	1.226503	C	-0.933283	3.554407	-0.647756
O	0.674535	-4.992254	1.923376	O	-0.823915	2.572216	-0.057721
**[$Ti_6Se_8(PMe_3)(CO)_2$]**							
**4***				**6***			
Ti	-4.271814	1.413784	13.031038	Ti	1.619535	8.849821	26.715743
Ti	-2.793378	-0.913511	12.863509	Ti	3.396744	6.666395	26.114824
Ti	-4.196489	-1.044796	15.400496	Ti	2.050019	6.163587	28.524375
Ti	-4.417722	-1.170505	10.610521	Ti	1.771285	6.869386	23.861659
Ti	-5.762667	-1.105129	13.108556	Ti	0.381394	6.367203	26.225033
Ti	-4.115862	-3.394502	13.027833	Ti	2.113298	4.192378	25.858739
Se	-5.962866	0.707579	11.209071	Se	-0.027297	8.377340	24.662603
Se	-2.338392	0.478862	10.649062	Se	3.609115	8.587407	24.353666
Se	-2.287199	0.819594	14.817944	Se	3.592033	8.308540	28.106424
Se	-5.940222	0.795385	14.825993	Se	-0.010574	7.972610	28.281950
Se	-6.040441	-3.036815	11.424420	Se	0.193386	4.724854	24.314309
Se	-5.823077	-2.877930	15.031437	Se	0.365553	4.436666	27.933532
Se	-2.269518	-2.529136	14.783175	Se	3.947802	4.766793	27.768737
Se	-2.443721	-2.819177	11.203415	Se	3.749977	5.065161	24.171171
H	-2.926690	1.858079	10.712419	H	2.860380	9.948337	25.016176
H	-3.165250	2.122248	14.679876	H	0.435783	11.910239	25.838093
C	-4.287742	3.510257	13.505601	C	1.338389	11.633010	25.253136
O	-3.657072	3.266974	12.409831	O	2.063675	10.673893	25.877329
O	-4.605243	4.464996	14.163621	O	1.644745	12.138992	24.204436
P	-4.341696	0.081477	17.740138	P	1.959034	6.929854	30.996052
C	-3.114978	-0.545089	18.958376	C	3.336318	6.212079	31.979377

C	-4.160645	1.900368	17.862698	C	2.019976	8.711814	31.408715
C	-5.955425	-0.249518	18.551104	C	0.454130	6.341051	31.868105
H	-3.216328	-1.633595	19.052996	H	3.315504	5.118740	31.888356
H	-2.104023	-0.322121	18.594826	H	4.289048	6.573319	31.572633
H	-3.261438	-0.078281	19.941915	H	3.256057	6.492902	33.038513
H	-3.141790	2.180146	17.569217	H	2.969570	9.129354	31.054597
H	-4.866521	2.381539	17.174759	H	1.199977	9.223289	30.890714
H	-4.352102	2.235447	18.890815	H	1.926553	8.854933	32.493594
H	-6.751491	0.187320	17.935442	H	-0.421709	6.814055	31.406691
H	-6.117648	-1.333058	18.608971	H	0.363008	5.254564	31.745925
H	-5.985796	0.184162	19.559781	H	0.493117	6.591761	32.936870
C	-3.797692	-5.025278	14.385111	C	2.319885	2.397130	27.026835
O	-3.639060	-5.902595	15.111123	O	2.428950	1.431959	27.642745
C	-4.362417	-2.578594	8.997732	C	1.795449	5.721497	22.036718
O	-4.354306	-3.326607	8.125363	O	1.804323	5.136690	21.048072
**[$Ti_6Se_8(CO)_3$]**							
**4***				**6***			
Ti	3.656101	-5.379007	45.370689	Ti	-7.867915	-9.140839	22.429553
Ti	5.190996	-7.584824	44.615846	Ti	-5.998960	-11.153983	21.477055
Ti	3.907956	-8.363193	47.080645	Ti	-7.231953	-12.065466	23.821207
Ti	3.483354	-7.330157	42.410389	Ti	-7.750147	-10.681765	19.337836
Ti	2.227043	-7.893194	44.891307	Ti	-9.004784	-11.621946	21.658216
Ti	3.916352	-10.072617	44.215089	Ti	-7.205841	-13.612487	20.881362
Se	1.924499	-5.691493	43.475215	Se	-9.563051	-9.398156	20.417020
Se	5.557394	-5.715962	42.770488	Se	-5.953157	-8.988223	19.977593
Se	5.718449	-6.305760	46.873498	Se	-5.805553	-9.779854	23.640515
Se	2.092530	-6.458677	47.012226	Se	-9.369534	-10.333394	23.916932
Se	1.927583	-9.374581	42.809263	Se	-9.206874	-12.940986	19.504732
Se	2.261642	-10.053362	46.325149	Se	-8.869191	-13.764810	23.029554
Se	5.813617	-9.612036	46.053960	Se	-5.304642	-13.235012	22.781012
Se	5.500434	-9.039300	42.536471	Se	-5.685960	-12.419039	19.289125
H	4.974373	-4.393472	43.175833	H	-6.697184	-7.764769	20.888983
H	4.774366	-5.039725	47.079016	H	-7.440030	-5.768635	23.342685
C	3.658375	-3.423149	46.306248	C	-6.960018	-6.037804	22.378726
O	4.144955	-3.401385	45.119431	O	-7.424878	-7.220208	21.908707
O	3.387145	-2.664139	47.193422	O	-6.144125	-5.359839	21.811621
C	3.956741	-7.545298	49.078808	C	-7.235370	-11.222838	25.814135
O	3.981263	-7.103190	50.136909	O	-7.233609	-10.778940	26.872149
C	4.251081	-11.966304	45.173014	C	-6.864150	-15.549510	21.773863
O	4.422458	-12.985092	45.674813	O	-6.680728	-16.586031	22.232432
C	3.496255	-8.312867	40.499565	C	-7.735541	-11.529935	17.345639
O	3.482859	-8.837713	39.478497	O	-7.731132	-11.957726	16.281246

Supplementary Figure 1. Dissociation Pathway of H_2 on pristine Ti_6Se_8 cluster (GGA-PBE/TZ2P)

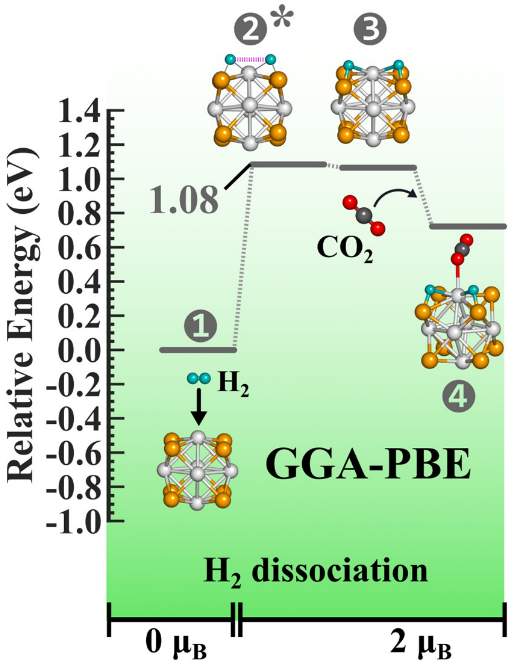

Supplementary Figure 2. The schematic of CO_2→HCOOH conversion on the $Ti_6Se_8(CO)_3$ cluster surface showing two possible reaction pathways (A and B)

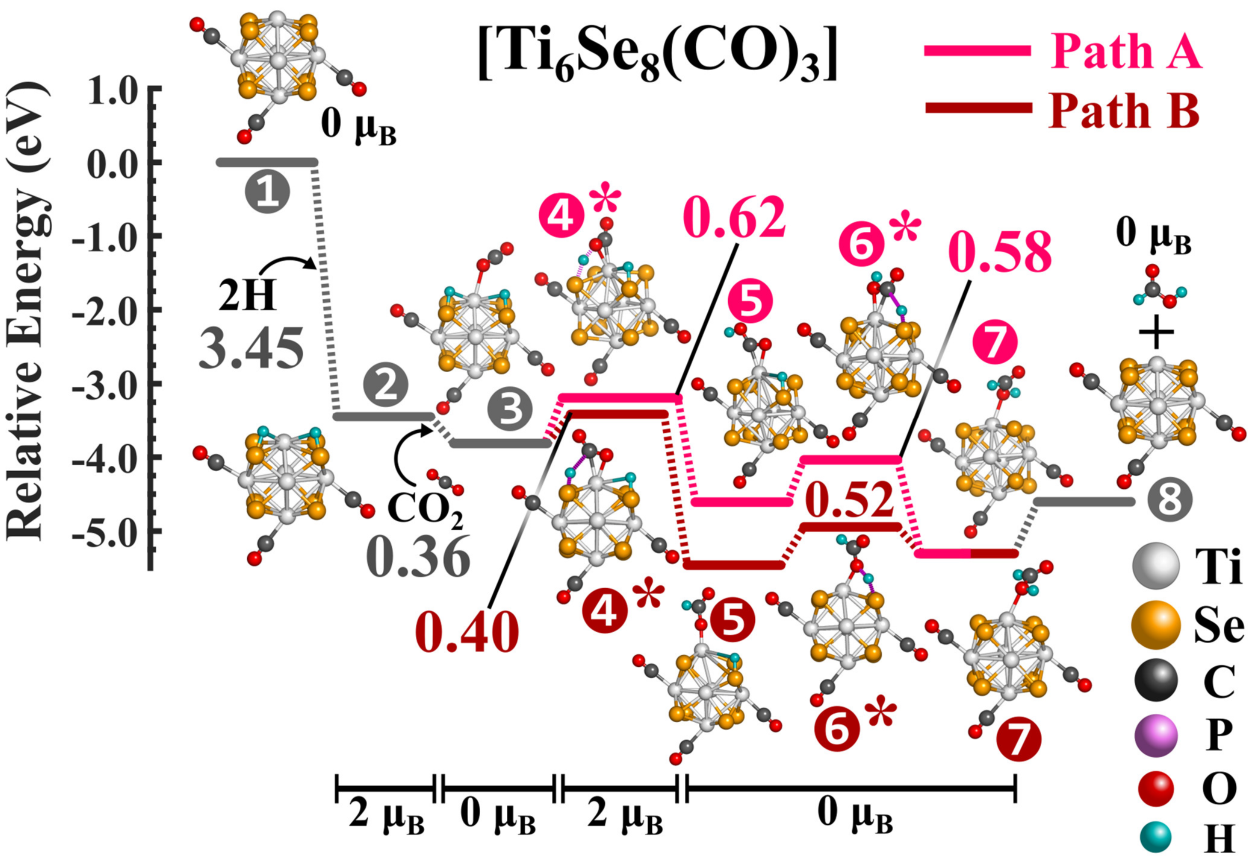

Supplementary Figure 3. Calculated reaction pathways of CO_2→HCOOH conversion on the (a) Ti_6Se_8 and (b)$Ti_6Se_8(CO)_3$ cluster in PBE0/TZ2P level of theory

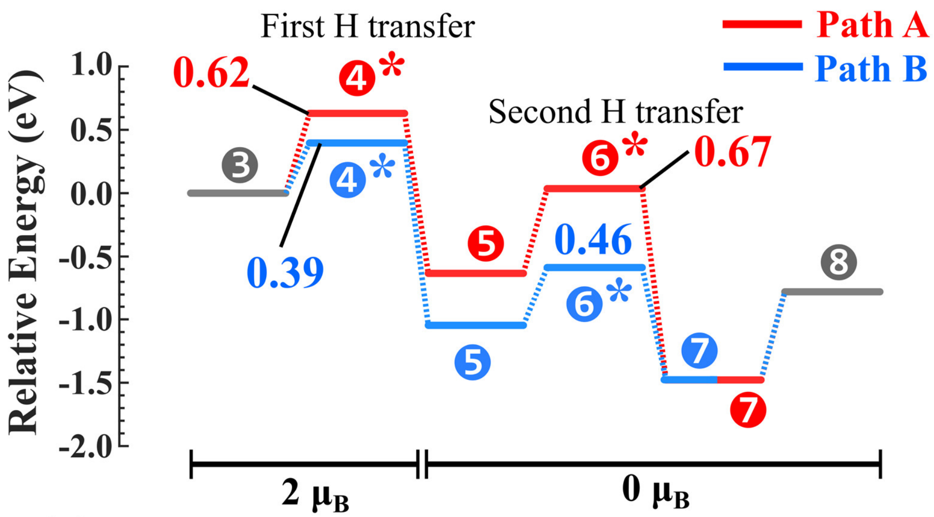

(a) Calculated (PBE0/TZ2P) reaction pathway of $CO_2 \rightarrow$ HCOOH conversion on Ti_6Se_8 cluster.

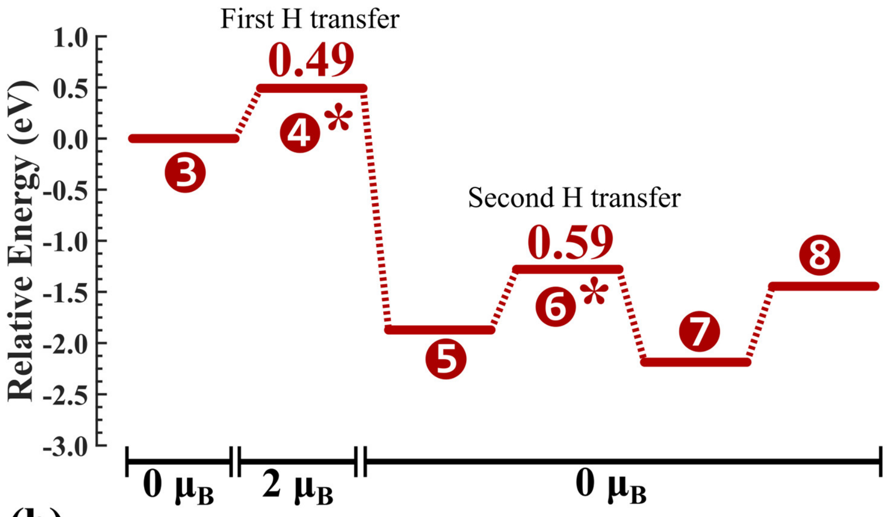

(b) Calculated (PBE0/TZ2P) reaction pathway of $CO_2 \rightarrow$ HCOOH conversion on $[Ti_6Se_8(CO)_3]$cluster.

Supplementary Figure 4. (a) Molecular orbital and (b) projected density of states diagram of intermediate 2 for the [$Ti_6Se_8(PMe_3)_{3-m}(CO)_m$], (m=0-3) and Ti_6Se_8 cluster.

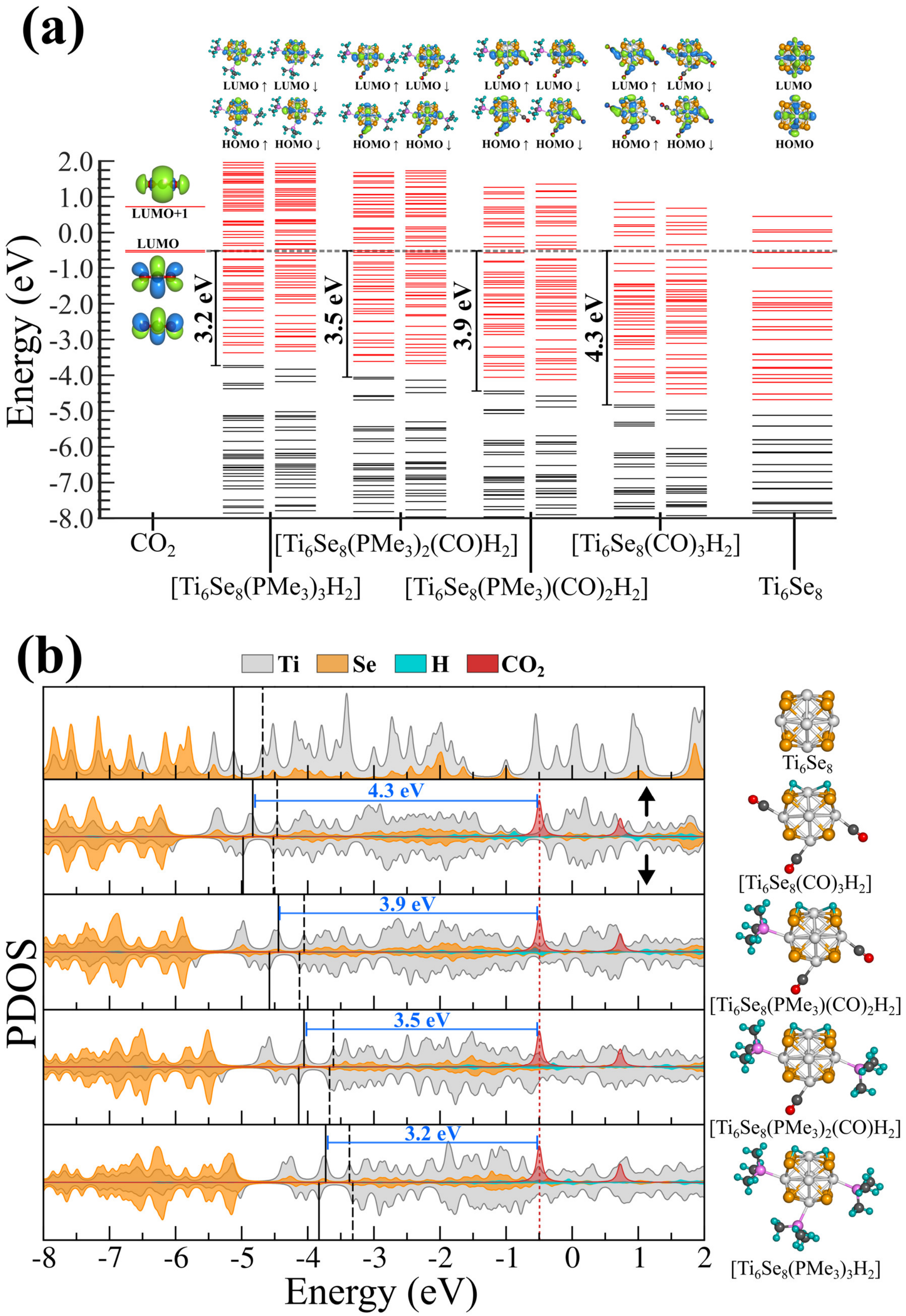

Supplement: Supplementary file 1 — Supplementary Information [file 42004_2023_851_MOESM1_ESM.pdf]
